# Genome-wide association study identifies PERLD1 as asthma candidate gene

**DOI:** 10.1186/1471-2350-12-170

**Published:** 2011-12-21

**Authors:** Ramani Anantharaman, Anand Kumar Andiappan, Pallavi Parate Nilkanth, Bani Kaur Suri, De Yun Wang, Fook Tim Chew

**Affiliations:** 1 Department of Biological Sciences, National University of Singapore, Singapore; 2 Department of Otolaryngology, National University of Singapore, Singapore

## Abstract

**Background:**

Recent genome-wide association studies (GWAS) for asthma have been successful in identifying novel associations which have been well replicated. The aim of this study is to identify the genetic variants that influence predisposition towards asthma in an ethnic Chinese population in Singapore using a GWAS approach.

**Methods:**

A two-stage GWAS was performed in case samples with allergic asthma, and in control samples without asthma and atopy. In the discovery stage, 490 case and 490 control samples were analysed by pooled genotyping. Significant associations from the first stage were evaluated in a replication cohort of 521 case and 524 control samples in the second stage. The same 980 samples used in the discovery phase were also individually genotyped for purposes of a combined analysis. An additional 1445 non-asthmatic atopic control samples were also genotyped.

**Results:**

19 promising SNPs which passed our genome-wide *P *value threshold of 5.52 × 10^-8 ^were individually genotyped. In the combined analysis of 1011 case and 1014 control samples, SNP rs2941504 in PERLD1 on chromosome 17q12 was found to be significantly associated with asthma at the genotypic level (*P *= 1.48 × 10^-6^, OR_AG _= 0.526 (0.369-0.700), OR_AA _= 0.480 (0.361-0.639)) and at the allelic level (*P *= 9.56 × 10^-6^, OR = 0.745 (0.654-0.848)). These findings were found to be replicated in 3 other asthma GWAS studies, thus validating our own results. Analysis against the atopy control samples suggested that the SNP was associated with allergic asthma and not to either the asthma or allergy components. Genotyping of additional SNPs in 100 kb flanking rs2941504 further confirmed that the association was indeed to PERLD1. PERLD1 is involved in the modification of the glycosylphosphatidylinositol anchors for cell surface markers such as CD48 and CD59 which are known to play multiple roles in T-cell activation and proliferation.

**Conclusions:**

These findings reveal the association of a PERLD1 as a novel asthma candidate gene and reinforce the involvement of genes on the 17q12-21 chromosomal region in the etiology of asthma.

## Background

Asthma is a highly complex disease of airway inflammation. The intricacies of asthma are exemplified in its diverse clinical characteristics in terms of its triggers, symptoms and presentation, as well as how the disease is understood at the molecular level. More than 180 genes have been found to be associated with asthma [1-3]. Recent genome-wide association studies (GWAS) for asthma have identified novel genes such as ORMDL3 [4], CHI3L1 [5] and DENND1B [6], and chromosomal regions such as 9q21.31 [7]. These as well as other known associations to genes PDE4D [8], TGFB1, IL1RL1, IL18R1, DPP10 [9], and regions of the highly replicated 5q31-33 and HLA gene clusters [10,11] have also been confirmed by other GWAS and meta-analyses of GWAS for asthma [12,13]. The identification of many of these novel asthma candidates has widened the scope of possible mechanisms involved in its etiology. The premise that genome-wide association studies when used as tools to elucidate the genetic variants associated with complex genetic diseases, will lead to greater understanding of the underlying biology is being exemplified in studies on asthma.

Pooled GWAS have been used as an alternative to large-scale GWAS, especially when performed as part of a two-stage study design to retain sufficient power to detect association in a particular sample size [14]. Since 2007, at least 15 separate pooled GWAS have shown significantly reasonable successes in identifying candidate genes for complex diseases such as autism [15], schizophrenia [16] and atopy [17]. We have also previously shown that pooled genotyping on the Affymetrix SNP6.0 platform is sufficiently accurate and reliable in a case-control study design [18].

**Figure 1:**
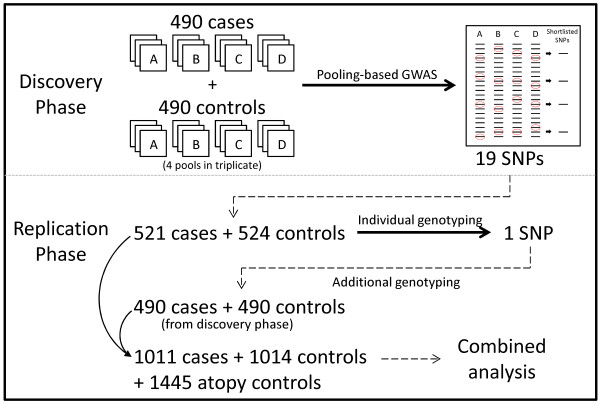
**Two-stage study design**. Four pools (A-D) of a total of 490 case and 490 control samples were created and subjected to a GWAS in the discovery phase. Each case-control pool set generated a list of SNPs passing the Bonferroni-corrected cut-off. 19 SNPs which consistently appeared in multiple lists were short-listed for replication. Following individual genotyping in 521 case and 524 control samples, 2 SNPs remained significantly associated. These SNPs were subsequently genotyped in the 490 case and 490 control samples to make up a total of 1011 case and 1014 control samples. An additional 1445 atopy control samples were also genotyped.

In this study, we have carried out a two-stage GWAS using pooled genotyping in the discovery stage which we followed up with individual genotyping in a replication sample set to replicate the associated signals (Figure 1). Additional genotyping was performed using the case and control samples used in the pools as well as in atopy control samples.

## Methods

### Samples

The case and control DNA samples used in this study were part of a larger cross-sectional epidemiological collection of unrelated adult ethnic Chinese students in the National University of Singapore who were recruited following standard protocols for written informed consent, over the span of four years. We have previously shown that there is negligible population stratification in samples from our study population [19]. Approval to conduct the study was obtained from the National University of Singapore Institutional Review Board (NUS-IRB Reference Code: 07-023 and NUS10-343). Recruitment was performed in compliance with the Helsinki Declaration.

Genomic DNA was extracted from buccal cells obtained from a mouthwash in 0.9% saline solution. In short, the buccal cells were pelleted and lysed; DNA was extracted using the phenol-chloroform phase-separation technique [20], purified by two washes in ethanol, with the DNA pellet resuspended in reduced Tris-EDTA buffer. The quality of the genomic DNA samples was assessed by gel electrophoresis and through the measurement of light absorption at 260 and 230 nm wavelengths. Samples were also quantified in quintuplicate using the PicoGreen (Molecular Probes Inc. Eugene, Oregon) dsDNA quantification reagent. We have also previously shown that these buccal cell-derived genomic DNA samples were amenable to Sanger sequencing [21] and individual genome-wide analysis on the Illumina and Affymetrix platforms [19].

Samples were subsequently stratified into case and control groups according to their disease status based on an International Study of Asthma and Allergies in Childhood (ISAAC)-derived survey questionnaire [22] and doctor-diagnosis. A skin-prick test for common local allergens in Singapore (*Dermatophagoides pteronyssinus*, *Blomia tropicalis*, *Elaeis guineensis *and *Curvularia lunata*) was also performed as a measure of their atopic status. We have previously shown that skin-prick reactivity to dust mites *Blomia tropicalis *and *Dermatophagoides pteronyssinus *were highly sensitive and specific indicators for allergic sensitization in Singapore [23]. Cases were defined as having asthma by a positive response to the question "Have you ever had asthma?", a doctor's diagnosis and a positive skin prick reaction (wheal diameter ≥ 3 mm) to either one of the dust mite allergens. In our sampling frame, 17% of the samples were considered to be cases. Conversely, a control answered "No" to the above question, did not have any symptoms of asthma and also showed no positive skin prick reactions to any of the allergens evaluated. About 17% of the sampled individuals were controls. A subset of the individuals completed lung function and peak flow reversibility test, as well as a complete physicians' assessment for asthma (data not shown). This is done to cross validate our diagnosis as well as classification of cases and controls. A third category of individuals without any symptoms of asthma or diagnosis but with positive skin-prick responses were selected as atopic controls. Table 1 summarizes the demographics of the population used in this study.

**Table 1:** Population demographics of sample sets used in GWAS and replication stages of the study

	Discovery		Replication		
**Classification^a^**	**Case**	**Control**	**Case**	**Control**	**Atopy Control**
**Age in years (Mean)^b^**	18-28 (21.7)		18-30 (20.3)		
**Disease^c^**	Asthma	No Asthma	Asthma	No Asthma	No Asthma
**Atopy^d^**	Yes	No	Yes	No	Yes
**Number of samples**	490	490	521	524	1445
**Male Gender (%)**	248 (50.4%)	132 (26.9%)	310 (59.5%)	158 (30.2%)	699 (48.4%)

a) Classification of samples in study population; b) Range in age of participants with mean age in brackets; c) Disease status; d) Atopy status as derived from a skin prick test.

### Pooling

As our cross-sectional sampling was performed in various stages over a four year period, at the time we carried out the GWAS, a total of 980 case and control samples were available for genotyping. These samples were selected for pooling because they fell within a 5% error margin in the replicate measurements by PicoGreen. 100 ng from each DNA sample was pooled together. Pooled DNA samples were re-quantified by PicoGreen to ensure accuracy of pooling. Four pools of varying size (from 90-160 samples per pool) were created leading up to 490 samples for each study group.

### Pooled GWAS

Genotyping of the pooled DNA samples on the Affymetrix Genome-Wide Human SNP Array 6.0 (Affymetrix. Santa Carla, California) platform was outsourced to a local service provider (Origen Laboratories Pte Ltd) in Singapore. Each of the eight pooled samples was genotyped in triplicate, resulting in the use of 24 microarray chips to genotype 490 case and 490 control samples.

### Allelotyping for pooled genotyping

Allele frequencies for the 906,600 SNPs on the Affymetrix SNP6.0 chip were estimated from probe intensities using the polynomial-based probe specific correction (PPC) [24] as previously described [18].

### Individual genotyping for replication

Genomic DNA samples were diluted to 50 ng/μl prior to being outsourced to the DNA Sequencing and Genomics Facility at the University of Utah for individual genotyping on the Illumina BeadXpress platform using the GoldenGate assay (Illumina, Inc. San Diego, California) and to the SNP Research Facility at the Washington University in St Louis for genotyping on the Sequenom platform using the MassARRAY iPlex assay (Sequenom, Inc. San Diego, California). An additional 521 case and 524 control samples (together with the initial 490 case and 490 control samples making up a total of 1011 cases and 1014 controls) were individually genotyped to replicate the observed associations from the discovery stage. An additional 1445 atopic samples without asthma were also genotyped (Table 1).

### Statistical analysis

Association analysis using the estimated allele frequencies from the pooled genotyping was performed in R [25] via a Z test and by generating a single-tail *P *value for significance.

The genotypes obtained in the replication stage were analysed using PLINK v1.07 [26] using the full model association tests (--model) and via Fisher's exact test (--fisher). This permitted us to test for association under the general, multiplicative, additive dominant and recessive genetic models [27]. *P *values from the tests for association under the different models are presented as *P_general_*, *P_multiplicative_*, *P_additive_*, *P_dominant _*and *P_recessive_*. *P_general _*and *P_multiplicative _*refer to the *P *values obtained from a Fisher's test performed on the genotype and allele counts respectively in cases and controls. *P_additive _*refers to the *P *value obtained from a Cochran Armitage Trend test for allele counts in cases and controls. *P_dominant _*refers to the *P *values obtained from a Fisher's test on the pooled genotype counts of the homozygous minor and heterozygous genotypes against the homozygous major genotype. *P_recessive _*refers to the *P *value from a Fisher's test on the genotype counts of the homozygous minor against the pooled counts of the heterozygous and homozygous major genotypes. Odds ratios are presented with the 95% confidence interval in parentheses. As we did not have any assumptions on the nature of the genetic association, association was tested for all the genetic models. However, only the general and multiplicative models were considered for determining whether an association was positive (or not). SNP call rates (--missing) and Hardy-Weinberg Equilibrium (--hardy) were also determined as quality control measures.

### Functional prediction of variants

The functional characteristics of tested SNPs were determined through the use of the online database F-SNP [28]. Splicing regulation sites were predicted using Human Splicing Finder [29].

## Results

### Pooled GWAS

In this study, a pooled GWAS was performed on 490 cases and 490 controls on the Affymetrix SNP6.0 platform (Table 1). Cases were defined as atopic individuals who have ever had asthma, while controls were neither atopic nor have ever had symptoms of asthma. As only ethnic Chinese individuals were selected to participate in this study, there was no bias or stratification based on ethnicity. Secondly, as the study samples were sourced from a cross-sectional sampling frame, selection bias was also avoided. We have also previously highlighted that our study population was notably similar to the Han Chinese population in Hapmap (CHB) and that there was negligible population stratification within samples from our cross-sectional epidemiological collection [19].

Four pools (A-D in Figure 1) were created within each study group, and each pool was genotyped in triplicate to account for potential errors due to pooled genotyping. SNP call rates were better than 96% while the concordance of probe intensities across replicates was higher than 97.5%. We have previously shown that estimated allele frequencies from pooled genotyping on the Affymetrix SNP 6.0 platform were highly comparable to actual allele frequencies obtained from individual genotyping on the same platform [18]. Estimated allele frequencies for SNPs were calculated from the measured probe intensities. The allele frequency estimates were averaged across the three replicates in each of the 4 pairs of case and control groups. These averaged estimated allele frequencies were compared between the study groups via a Z-test to generate 4 sets of *P *values.

Of the 906,600 SNPs on the Affymetrix SNP 6.0 array, 185,875 non-variable and 37,376 non-autosomal SNPs SNPs were discarded. The remaining 683,349 SNPs were sorted according to their *P *values to generate 4 SNP lists. As these *P *values were solely used to rank the SNPs for replication and weren't considered to be indicative of the strength of association, SNPs were selected for further study using a standard Bonferroni *P *value cut-off of 5.52 × 10^-8 ^(α = 0.05 divided by 906,600 SNPs). Furthermore, as only estimated allele frequencies were available, the obtained *P *values could merely be considered indicative of association in a multiplicative allelic model. Common SNPs (> 5% based on Hapmap CHB) which were ranked below this cut-off were short listed from the 4 case-control comparisons. 19 SNPs were found to consistently appear in at least 2 of these SNP lists; 18 of which were consistently ranked below the cut-off in two case-control comparisons while 1 in three case-control comparisons (Table 2). These 19 SNPs were shortlisted for replication.

**Table 2:** Genotyping results of 19 SNPs in discovery phase

SNP^a^	Gene^b^	CHB MAF^c^	*P*_Pool A_^d^	*P*_Pool B_^e^	*P*_Pool C_^f^	*P*_Pool D_^g^	No. of pools^h^
**rs2941504**	PERLD1	0.456	7.82 × 10^-6^	*3.06 × 10^-8^	*3.94 × 10^-13^	*1.78 × 10^-10^	3
**rs6036630**	-	0.189	*2.64 × 10^-9^	*3.76 × 10^-9^	2.86 × 10^-2^	4.51 × 10^-1^	2
**rs2304368**	R3HDM1	0.056	*1.63 × 10^-11^	*9.58 × 10^-9^	1.55 × 10^-5^	2.79 × 10^-2^	2
**rs12590520**	-	0.389	1.00	*1.46 × 10^-9^	*8.66 × 10^-9^	5.50 × 10^-1^	2
**rs11895665**	-	0.267	*2.23 × 10^-9^	*6.53 × 10^-9^	2.08 × 10^-1^	1.30 × 10^-1^	2
**rs10520302**	GPM6A	0.378	*1.21 × 10^-8^	1.16 × 10^-3^	8.26 × 10^-1^	*2.31 × 10^-9^	2
**rs11225634**	DYNC2H1	0.122	5.03 × 10^-1^	*1.57 × 10^-8^	9.40 × 10^-2^	*2.97 × 10^-14^	2
**rs7930326**	-	0.200	*8.92 × 10^-9^	5.12 × 10^-2^	*2.06 × 10^-10^	8.89 × 10^-1^	2
**rs12601246**	SMYD4	0.057	*1.07 × 10^-9^	6.89 × 10^-2^	7.69 × 10^-1^	*1.60 × 10^-8^	2
**rs4738955**	NKAIN3	0.081	*5.78 × 10^-15^	3.36 × 10^-1^	*2.11 × 10^-13^	1.82 × 10^-1^	2
**rs8133478**	RUNX1	0.078	1.94 × 10^-1^	*9.57 × 10^-9^	1.35 × 10^-1^	*6.39 × 10^-11^	2
**rs9292197**	PDE4D	0.189	7.97 × 10^-1^	9.32 × 10^-1^	*1.05 × 10^-10^	*1.19 × 10^-8^	2
**rs11904654**	LTBP1	0.478	5.85 × 10^-1^	*3.39 × 10^-9^	5.89 × 10^-1^	*1.16 × 10^-8^	2
**rs11948993**	-	0.341	*1.15 × 10^-9^	3.27 × 10^-5^	*6.49 × 10^-9^	3.60 × 10^-1^	2
**rs6788482**	-	0.341	2.20 × 10^-1^	*9.58 × 10^-9^	8.17 × 10^-1^	*5.29 × 10^-9^	2
**rs6793110**	LRIG1	0.056	*2.43 × 10^-11^	7.26 × 10^-6^	*6.99 × 10^-9^	4.82 × 10^-5^	2
**rs10917042**	USP48	0.433	*2.75 × 10^-11^	1.60 × 10^-1^	5.73 × 10^-2^	*1.50 × 10^-9^	2
**rs2268451**	TSHR	0.279	2.84 × 10^-1^	*2.47 × 10^-8^	*5.52 × 10^-9^	1.16 × 10^-1^	2
**rs5993853**	TXNRD2	0.156	4.33 × 10^-1^	*6.11 × 10^-9^	2.44 × 10^-1^	*1.30 × 10^-8^	2

a) The SNP rsID; b) Gene name or hyphen if intergenic; c) SNP minor allele frequency in Hapmap CHB population; d)-g) *P *values obtained from a Z-test of estimated allele frequencies of case vs control in pools A-D (These *P *values were solely used to rank the SNPs for replication and weren't considered to be indicative of the strength of association.); h) The number of pool sets in which SNP was ranked below a Bonferroni-corrected *P *value of 5.52 × 10^-8 ^(*P *values indicated with a *)

### Replication

These 19 SNPs were individually genotyped in 1045 samples consisting of 521 case and 524 control samples (Table 1) in the replication stage. Genotyping success rate was more than 97% for all SNPs. None of the genotyped SNPs showed any major deviation (*P *value < 1 × 10^-3^) from Hardy Weinberg Equilibrium. Association with asthma was tested under the general and multiplicative genetic models [27] to account for the genotypic and allelic effect of these 19 SNPs (Table 3). Since the gender ratio was skewed in our sample set, we tested for any gender-related effects by performing the association tests in male and female samples separately. No gender-specific associations were found (data not shown).

**Table 3:** Genotyping results of 19 SNPs in replication stage, sorted by *P *values under general model.

SNP^a^	Variation^b^	Chr^c^	Gene^d^	GWAS^e^	General^f^	Multiplicative^g^	OR (95% C.I.)^h^
**rs2941504**	(G/A)	17q12	PERLD1	3	2.60 × 10^-3^	9.26 × 10^-3^	0.784 (0.653-0.941)
**rs6036630**	(T/C)	20p11.21	-	2	8.58 × 10^-3^	6.33 × 10^-2^	1.397 (0.986-1.980)
**rs2304368**	(A/C)	2q21.3	R3HDM1	2	7.06 × 10^-2^	7.70 × 10^-2^	1.808 (0.940-3.475)
**rs12590520**	(T/C)	14q24.3	-	2	2.07 × 10^-1^	1.39 × 10^-1^	1.244 (0.944-1.641)
**rs11895665**	(T/C)	2q37.3	LRRFIP1	2	2.47 × 10^-1^	2.76 × 10^-1^	1.194 (0.879-1.620)
**rs10520302**	(T/C)	4q34.2	GPM6A	2	2.74 × 10^-1^	9.44 × 10^-1^	0.987 (0.749-1.301)
**rs11225634**	(G/T)	11q22.3	DYNC2H1	2	3.55 × 10^-1^	2.28 × 10^-1^	1.375 (0.855-2.212)
**rs7930326**	(T/C)	11q23.1	-	2	4.83 × 10^-1^	2.40 × 10^-1^	0.811 (0.582-1.128)
**rs12601246**	(T/C)	17p13.3	SMYD4	2	5.67 × 10^-1^	5.14 × 10^-1^	1.170 (0.764-1.794)
**rs4738955**	(A/G)	8q12.3	NKAIN3	2	5.76 × 10^-1^	5.89 × 10^-1^	0.847 (0.496-1.445)
**rs8133478**	(G/A)	21q22.3	RUNX1	2	6.10 × 10^-1^	5.28 × 10^-1^	0.844 (0.512-1.391)
**rs9292197**	(C/T)	5q11.2	PDE4D	2	7.48 × 10^-1^	8.49 × 10^-1^	1.043 (0.717-1.516)
**rs11904654**	(G/T)	2p22.3	LTBP1	2	8.09 × 10^-1^	6.90 × 10^-1^	0.941 (0.725-1.221)
**rs11948993**	(C/T)	5p15.31	-	2	8.29 × 10^-1^	5.94 × 10^-1^	0.916 (0.679-1.235)
**rs6788482**	(A/G)	3q13.13	-	2	8.39 × 10^-1^	1.00	1.009 (0.746-1.364)
**rs6793110**	(A/G)	3p14.1	LRIG1	2	8.78 × 10^-1^	6.50 × 10^-1^	0.891 (0.570-1.395)
**rs10917042**	(C/T)	1p36.12	USP48	2	8.84 × 10^-1^	6.81 × 10^-1^	0.939 (0.718-1.229)
**rs2268451**	(G/T)	14q31.1	TSHR	2	8.95 × 10^-1^	7.49 × 10^-1^	0.944 (0.689-1.292)
**rs5993853**	(T/C)	22q11.21	TXNRD2	2	1.00	7.72 × 10^-1^	0.892 (0.503-1.579)

a) The SNP rsID; b) The polymorphism in the format (minor allele/ancestral allele); c) Chromosomal location; d) Gene name or hyphen if intergenic; e) Number of case-control comparisons in which SNP was ranked significantly in pooled GWAS; f) *P *value obtained from a Fisher's test for a general genetic model; g) *P *value obtained from a Fisher's test for a multiplicative genetic model; h) Allelic odds ratio with 95% confidence interval, calculated from allele counts using the minor allele as the reference.

After accounting for multiple testing, one SNP was found to be significant with a *P *value less than the Bonferroni-corrected value of 2.63 × 10^-3 ^(0.05/19 SNPs). This SNP (rs2941504) falls within the gene PERLD1 on chromosome 17q12 and is coding synonymous. It showed positive association to asthma in the replication stage (*P*_general _= 2.60 × 10^-3^). While the minor G allele was present at a lower frequency in cases (MAF_case _= 0.305) than in controls (MAF_control _= 0.359) with an odds ratio of 0.784 (0.653-0.941), it was not significantly associated at the allelic level (*P*_multiplicative _= 9.26 × 10^-3^) after accounting for multiple testing.

The SNP rs2941504 was subsequently genotyped in the 490 case and 490 control samples used in the discovery phase, to improve on the sample numbers and in an attempt to obtain genotype data to perform a combined analysis. This permitted association testing in a total of 1011 case and 1014 control samples. Given this sample size, the frequency of the minor allele in our genotyped population (MAF = 0.350), the prevalence of asthma in our cross-sectional sampling frame (17%), and a genome-wide significance level (α = 5 × 10^-8^), we had 80% power, based on joint analysis in a two-staged study design [30], to detect associations with odds ratios greater than 1.4 [31]. While either one of the stages of our study would not have been individually sufficiently powered, the combined analysis from both stages did have sufficient power to say that the discovered associations were indeed true.

The coding SNP, rs2941504, in the PERLD1 gene showed strong association (*P_general _*= 1.48 × 10^-6^) to asthma (Table 4) in the combined analysis. The genotypic odds ratio of the minor GG genotype as the reference revealed that it had a moderate effect of between 0.480 and 0.526 over the AA and AG genotypes respectively. The presence of the minor G allele at a significantly (*P_multiplicative _*= 9.56 × 10^-6^) higher frequency in controls than in cases (MAF_controls _= 0.381, MAF_cases _= 0.315) suggested that it conferred some protection against asthma (OR: 0.745 (0.654-0.848)). The effect of the minor allele is seen more pronounced in the recessive genetic model with a *P_recessive _*value of 4.34 × 10^-7 ^with odds ratio of 0.503 (0.384-0.658). As there was no major deviation from Hardy Weinberg Equilibrium, the allelic association as tested by a Cochran-Armitage trend test generated a *P *value (*P*_additive _= 1.25 × 10^-5^) which was similar to that obtained from a Fisher's test on the allele counts.

**Table 4:** Additional genotyping at 17q12 to localize association signal

SNP^a^	Variation^b^	Gene^c^	Case^d^	Control^e^	Multiplicative^f^	Regression^g^
**rs12601930**	(T/C)	PPP1R1B	0.188	0.179	5.59 × 10^-1^ 1.061 (0.876-1.284)	5.73 × 10^-1^ 0.944 (0.771-1.155)
**rs9532**	(T/C)	PPP1R1B	0.001	0.001	1.00 1.007 (0.063-16.118)	9.54 × 10^-1^ 1.085 (0.066-17.84)
**rs1877031**	(T/C)	STARD3	0.346	0.401	2.96 × 10^-3^ 0.792 (0.679-0.924)	1.96 × 10^-1^ 1.419 (0.835-2.412)
**rs876493**	(T/C)	PNMT	0.266	0.305	2.37 × 10^-2^ 0.825 (0.700-0.973)	4.50 × 10^-1^ 1.111 (0.846-1.460)
**rs2941504**	(A/G)	PERLD1	0.305	0.359	9.56 × 10^-6^ 0.745 (0.654-0.848)	NA
**rs1136201**	(G/A)	ERBB2	0.097	0.123	3.03 × 10^-2^ 0.768 (0.606-0.974)	5.89 × 10^-1^ 0.930 (0.715-1.210)
**rs1058808**	(G/C)	ERBB2	0.325	0.389	7.81 × 10^-4^ 0.756 (0.644-0.889)	9.00 × 10^-2^ 0.652 (0.398-1.069)

The genotyped SNPs are sorted according to chromosomal position at 17q12 (not shown).

a) The SNP rsID; b) The polymorphism in the format (minor allele/ancestral allele); c) Gene name; d) & e) Minor allele frequencies in case and control samples respectively; f) *P *value obtained from a Fisher's test for a multiplicative genetic model together with allelic odds ratio and 95% confidence interval calculated from allele counts using the minor allele as the reference; g) *P *value obtained from a logistic regression analysis of allelic association using PERLD1 SNP rs2941504 as a covariate together with odds ratio and 95% confidence interval from the regression.

### Localizing the association signal

**Figure 2:**
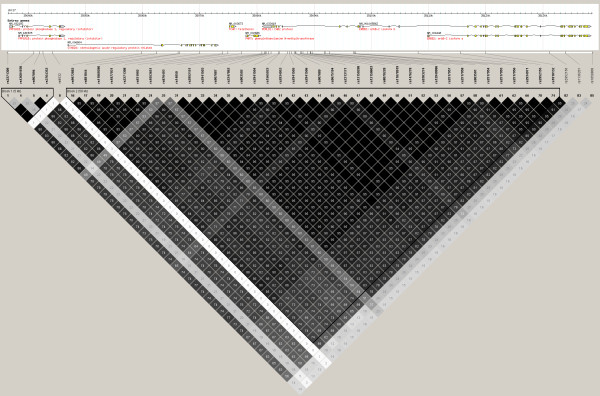
**Linkage disequilibrium pattern in 100 kb spanning SNP rs2941504 on PERLD1**. LD pattern generated using genotypes for 139 Hapmap CHB samples, in Haploview. SNPs with MAF lower than 0.05 were excluded. Linkage disequilibrium between any 2 SNPs is indicated in r^2 ^values in the boxes; higher r^2 ^values correspond to darker shading of the box, a black box without any number indicates perfect linkage (r^2 ^= 1.00).

To confirm that this association on 17q12 was indeed at PERLD1 and not at other adjacent genes, tagging was performed in the 100 kb flanking SNP rs2941504 to identify additional SNPs for genotyping. Tagging performed using the Hapmap CHB population as a reference with r^2 ^and MAF cut-offs of 0.8 and 0.05 respectively gave 7 SNPs (including rs2941504) to represent the region. The 6 additional SNPs were genotyped across 1011 case and 1014 controls (Table 4). After accounting for multiple testing, SNPs rs1877031 on STARD3 and rs1058808 on ERBB2 were found to have *P *values less than 8.33 × 10^-3 ^(0.05/6 SNPs). To determine whether these additional associations were influenced by the strong linkage disequilibrium in the region to PERLD1 (Figure 2), logistic regression using SNP rs2941504 as a covariate was performed in PLINK (--logistic --condition). None of the SNPs remained significant in the regression analysis (Table 4). This confirmed that genome-wide association signal at 17q12 was indeed due to PERLD1, and not any other gene in the region.

### Genotyping of Atopic Controls

The SNP rs2941504 was also genotyped in an additional 1445 atopic individuals without asthma (Table 1). To study the effect this SNP had on asthma without considering the effects of atopy, association was tested between the 1011 case and 1445 atopy control samples. Association was detected at the genotypic (*P_general _*= 2.25 × 10^-2^) and allelic (*P_multiplicative _*= 6.97 × 10^-3^) level with an allelic odds ratio of 0.845 (0.747-0.954). Similarly, the effect this SNP had at the level of atopy was also measured by testing for association between 1445 atopy controls and 1014 non-asthmatic non-atopic controls. Positive association was detected at the genotypic (*P_general _*= 1.96 × 10^-3^) as well as the allelic levels (*P_multiplicative _*= 3.87 × 10^-2^) with an allelic odds ratio of 0.882 (0.783-0.993) (Table 5).

**Table 5:** Genotyping results for rs2941504.

	Genetic Model	Genotype/Allele^d^	Affected^e^	Unaffected^f^	P value^g^	OR	95% CI^h^
	General	GG/AG/AA	93/449/467	170/432/410	1.48 × 10^-6^	0.526 0.480	(0.396-0.700) (0.361-0.639)
	Multiplicative	G/A	635/1383	772/1252	9.56 × 10^-6^	0.745	(0.654-0.848)
**1011** **vs**	Additive	G/A	635/1383	772/1252	1.25 × 10^-5^	0.745	(0.654-0.848)
**1014^a^**	Dominant	GG+AG/AA	542/467	602/410	9.25 × 10^-3^	0.790	(0.663-0.943)
	Recessive	GG/AG+AA	93/916	170/842	4.34 × 10^-7^	0.503	(0.384-0.658)

**1011** **vs**	General	GG/AG/AA	93/449/467	163/644/570	2.25 × 10^-2^	0.818 0.696	(0.617-1.085) (0.525-0.924)
**1445^b^**	Multiplicative	G/A	635/1383	970/1784	6.97 × 10^-3^	0.845	(0.747-0.954)

**1445** **vs**	General	GG/AG/AA	163/644/570	170/432/410	1.96 × 10^-3^	0.643 0.690	(0.502-0.823) (0.537-0.885)
**1014^c^**	Multiplicative	G/A	970/1784	772/1252	3.87 × 10^-2^	0.882	(0.783-0.993)

a) Genotyping results for 1011 case versus 1014 control samples comparison; b) Genotyping results for 1011 case versus 1445 atopic samples comparison; c) Genotyping results for 1445 atopic versus 1014 control samples comparison; d) Order of genotype and/or allele counts listed in columns e) and f); g) P values from a Fisher's test using a 3 × 2 table for General genetic model, a 2 × 2 table for Multiplicative, Dominant or Recessive models, and P value from a Cochran Armitage Trend test for Additive model; h) Odds ratio and 95% confidence interval calculated for the AG and AA genotypes respectively using GG as the reference under the General model, for the A allele using G as the reference under the Multiplicative and Additive models, for the AA genotype using GG and AG as the reference under the Dominant model, and for the AG and AA genotypes using GG as the reference under the Recessive model.

## Discussion

At the advent of genome-wide association studies (GWAS), investigators saw it as a "magic bullet" to deciphering the intricacies of complex diseases. While the initial acceptance was slow due to the massive investment normally available only to large-scale consortia, early successes [32], advances in technology and highly reduced prices have enabled more than 800 GWAS to be conducted on more than 400 diseases to date [33]. For asthma, recent reviews [34-36] have summarized the contribution of GWAS to the study of genetics of this disease. While different GWAS have identified numerous susceptibility variants, the results from these studies have not been able to fully explain the heritability of asthma and allergy. While epistasis, gene-environment interactions, rare and un-detected/un-known variants have been suggested to account for the "missing" heritability, none of these discount the ability of the GWAS to highlight important genes that are likely to be involved in susceptibility to the disease.

This paper presents a genome-wide case-control association study performed using pooled genotyping to study asthma in an ethnic Chinese population. Giampaolo Ricci *et al*. very recently also published a pooling-based GWAS on asthma. They tested 269 children of European descent comparing asthmatics and non-asthmatics with rhino-conjunctivitis [37]. The strongest association detected was for a SNP on the C5 gene at a *P *value of 1.1 × 10^-3^. Our study improves on this with a larger sample size in the discovery as well as replication stages, which resulted in associations with higher levels of significance. Observations from our GWAS were that multiple SNPs throughout the genome (Table 2) passed the standard genome-wide threshold of 5.52 × 10^-8 ^in multiple case versus control comparisons. Replication in a larger sample set via individual genotyping revealed that the strongest association signal was at PERLD1 on chromosome 17.

The 17q chromosomal region has been linked with asthma and allergy, via both linkage and candidate gene based studies in the past. Early genome-wide linkage studies identified associations of the 17q12-23 region to asthma and atopy in European populations [38,39]. Two recent meta-analyses of genome-wide linkage screens for asthma and its associated phenotypes have also indicated the significance of the 17q12-q24 region with allergic asthma and associated sub-phenotypes [40,41]. Candidate gene-based studies have identified genes such as CCL2 and CCL5 (17q11-q12), CCL11 and ITGB3 (17q21), and ACE (17q23), which have been found to be associated and well replicated in multiple populations [1,2]. The first GWAS for childhood asthma identified ORMDL3 (and GSDMB) also present in the 17q21 region [4]. This association has also been well replicated in multiple populations including Scottish, Northern Europeans, North Americans of European ancestry, African, Australian, French-Canadian, Mexicans, Puerto Ricans, and Koreans [12,13,42-46].

The PERLD1 SNP (rs2941504), whose association we identified and replicated, was also found to be associated with asthma in a number of other studies. In one of the studies which replicated the association on 17q21 to asthma [44], a GWAS was performed on 4917 case and 34,589 control samples from six European and one Korean populations. In this study, the presence of the A allele of SNP rs2941504 was reported to be significantly associated with asthma with *P *value of 1.2 × 10^-8 ^and odds ratio of 1.29; this association remained significant (*P *= 3.2 × 10^-2^) even after adjusting for the effect of the originally associated ORMDL3 variant (*P *= 1.56 × 10^-7^). In another GWAS on asthma [10], 473 cases, 1892 population controls and 363 phenotyped controls of European descent were genotyped. While the strongest associations reported were to RAD50 (*P *= 3.04 × 10^-7^) and HLA-DQB1 (*P *= 9.55 × 10^-6^), two SNPs within PERLD1 (rs1565922 and rs2941503) also had *P *values which were less than 0.05 (*P *= 4.63 × 10^-4 ^and *P *= 6.46 × 10^-4^). Most recently, a large-scale, consortium-based GWAS of asthma reiterated the significance of the 17q region in childhood asthma through the genotyping of more than 26,000 individuals [12] of European descent. In this latest study, genes such as ORMDL3 and GSDMB expectedly showed the strongest significance to childhood asthma (random-effect *P *values of 5.24 × 10^-21 ^and 6.45 × 10^-23 ^respectively). Interestingly, 4 SNPs within PERLD1 (including rs2941504 which we had found significant) were also presented with highly significant *P *values ranging from 1.26 × 10^-7 ^to 1.74 × 10^-10 ^with odds ratios around 0.84 (0.79-0.90). While none of the above mentioned studies explicitly mentioned PERLD1 being associated with asthma, the indicated *P *values were either listed in tables within the main text or in supplementary material. While many studies replicating the association of the 17q region to asthma have been published, association with SNPs in PERLD1 have only appeared in the above mentioned three. This suggests that despite the proximity to ORMDL3 and GSDMB, the association at PERLD1 might possibly be independent from them. In this study, the genome-wide signal whose association we confirmed was indeed at PERLD1 (Table 4) rather than anywhere else in the region despite the strong linkage disequilibrium to adjacent genes on 17q12 (Figure 2). In all, the results from these different studies rather effectively replicate the association that we independently discovered through our own GWAS.

There has been considerable evidence to suggest that allergic sensitization does not correlate well with allergic disease presentation [47,48]. As such, many genetic studies choose to use case-control definitions based purely on doctor diagnosis or self-reported symptomology without taking into account the atopic condition. This is done to exclude any interference from genes involved in the general mechanisms of allergy. However, we feel that not accounting for allergic sensitization detracts the involvement of immunological mechanisms from the etiology of allergic disease. We might also be discrediting the possibility of atopic individuals developing asthma later on. While this was the impetus for our use of non-asthmatic, non-atopic controls in our study, we also genotyped an additional 1445 non-asthmatic atopic control samples. Doing so allowed us to see the effect our significant SNP had on atopy itself as well asthma while disregarding the effects of atopy. Positive associations at *P *values less than 0.05 were detected in both the asthma versus atopy and atopy versus control comparisons. This suggested that there were indeed significant differences between the diseased, healthy and "intermediate" conditions. This was not unexpected, as an association to allergic asthma would likely involve both an 'asthmatic' and an 'allergy' component. However, the difference in the *P *values obtained from a case versus control comparison (*P_general _*= 1.48 × 10^-6^), and those obtained from a case versus atopy (*P_general _*= 2.25 × 10^-2^) or atopy versus control (*P_general _*= 1.96 × 10^-3^) comparisons are suggestive that the association is likely to allergic asthma and not to either of the conditions individually (Table 5).

**Figure 3:**
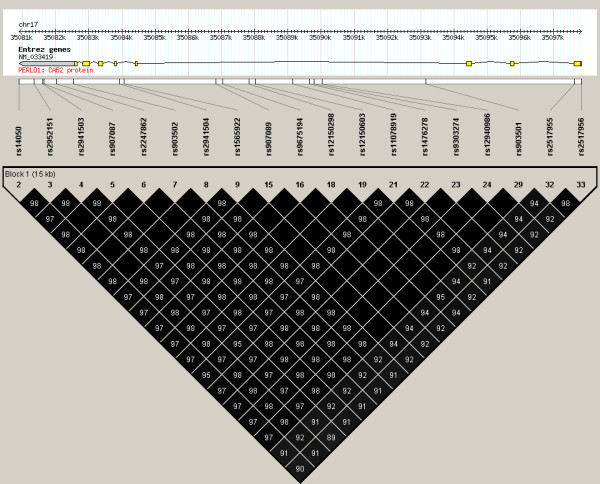
**Linkage disequilibrium pattern in PERLD1**. LD pattern generated using genotypes for 139 Hapmap CHB samples, in Haploview. SNPs with MAF lower than 0.05 were excluded. Linkage disequilibrium between any 2 SNPs is indicated in r^2 ^values in the boxes; higher r^2 ^values correspond to darker shading of the box, a black box without any number indicates perfect linkage (r^2 ^= 1.00).

The PERLD1 gene is observed to be in complete linkage disequilibrium based on the Hapmap CHB reference population (Figure 3). In other words, rs2941504 was able to tag all the variation in the whole gene. This suggests that genotyping other variants within the gene would produce similar associations. Furthermore, any functional variants within the gene could possibly lead to variation in gene function. Sequence motif-based prediction of functionality of the SNPs within PERLD1 (based on Hapmap CHB) revealed that most of the SNPs were likely to be involved in splicing regulation, either as exon-splicing enhancer or exon-splicing silencer recognition sites (data not shown). Alternative splicing of the mRNA due to the effects of any combination of these SNPs can possibly result in structural and/or functional diversification of PERLD1.

PERLD1, originally discovered as PER1 in yeast, was found to be involved in the lipid remodeling of glycosylphosphatidylinositol (GPI) anchors [49]. GPI-anchors are glycolipid moieties which are added on to the C-terminus of proteins during post-translational modification. Once attached, the GPI-anchor allows cell-surface proteins such as CD48 and CD59 to be attached onto the cell membrane [50]. The importance of GPI-anchors in cell-signaling has been observed in mammalian T-cells where cell-surface molecules which were displayed via GPI-anchors lost their ability to stimulate T-cell activation and proliferation. This occurred when either the proteins themselves were inhibited by specific antibodies or their ability to be displayed on the cell surface was affected through the inhibition of GPI-anchor attachment to their c-terminus [51]. CD48 was found to be a critical effector molecule in cell adhesion, pathogen recognition, and activation and regulation of various immunoregulatory pathways [52-55]. CD59, the principal ligand for CD2, has been shown to enhance T-cell activation and proliferation following cross-linking with CD2 [56]. Both these GPI-anchored markers were shown to be involved in proliferation of T-cells in patients suffering from bone marrow failure [57] and paroxysmal nocturnal haemoglobinuria [58]. The ability to regulate the activity and proliferative ability of T-cells through the control of GPI-anchor attachment suggest PERLD1's involvement in the inflammatory aspect of asthma.

We understand that the main limitation of this study is the pooling approach we took in the discovery phase. The use of pooled genotyping introduces errors due to array variation [59] as a result of differential hybridization of samples within the pool to the various probes of the array. Secondly, even though we had previously shown that the estimated allele frequencies obtained from pooled genotyping highly resemble the actual allele frequencies of the samples within the pool [18], we still suspected that they might represent the relative frequency of the allele within the pooled samples rather than the actual allele frequency of the samples. This could possibly result in an inflation of the differences in allele frequencies between case and control pools, and hence inflated *P *values and potential false positives. Lastly, the use of pooled samples means that actual genotypes will not be available thus preventing more detailed analysis of the nature or model of association.

We solved these issues by firstly genotyping each pool in triplicate and averaging across the replicates to account for the error due to the array itself. Secondly, we used the obtained *P *values only to rank the SNPs in terms of relative differences in allele frequencies within each pool pair; these *P *values were not considered to be indicative of strength of association. The commonly employed Bonferroni-corrected genome-wide cut-off was used to narrow down a list of potential hits. To filter out false positives, we compared the 4 separate lists of SNPs obtained from separate analysis of the 4 sets of pools (A-D), and shortlisted SNPs which appeared in 2 or more lists. We hypothesized that SNPs which passed the cut-off in more case-control comparisons were more likely to be truly associated and not just false positive hits. True enough, the association we are able to replicate (SNP rs2941504 on PERLD1) was the only one to appear in 3 out of the 4 sets of shortlisted SNPs; the *P *value in the 4^th ^pool set (Pool A, Table 2) was also relatively lower than any of the other *P *values of the remainder 18 shortlisted SNPs. Furthermore, it was the only SNP out of the 19 shortlisted ones which remained significant in the replication stage, suggesting that all the other SNPs were likely to have been false positives. We also individually genotyped the samples used in the original pools to permit a combined analysis with the replication sample set which allowed the elucidation of the nature of and identification of the best fit model to explain the observed association. This allowed us to prove that the association at rs2941504 was indeed real, and not just an artefact of pooled genotyping in the discovery phase.

## Conclusions

In this study, we have overcome the limitations of using pooled samples in the discovery phase, to identify a novel asthma gene whose association we replicated in an independent sample set; this association can also be corroborated by data from other studies. While it is the ORMDL3 and GSDMB genes in the 17q21 region that have been repeatedly linked with asthma in numerous other populations, it is not unreasonable to expect the immediately adjacent PERLD1 in the 17q12 region to also be directly associated with asthma in a Singapore Chinese population which has yet to be studied for the disease. The association of this gene offers new insights into the mechanisms of genetic susceptibility to asthma. While further studies are needed to investigate its functional relevance to the disease, our identification of PERLD1 as an asthma gene reinforces the importance of chromosome 17q12 in the etiology of the disease.

## Abbreviations used

**ACE**: angiotensin I converting enzyme; **C5**: complement component 5; **CCL11**: chemokine (C-C motif) ligand 11; **CCL2**: chemokine (C-C motif) ligand 2; **CCL5**: chemokine (C-C motif) ligand 5; **CD2**: Cluster of differentiation 2; **CD48**: Cluster of differentiation 48; **CD59**: Cluster of differentiation 59; **CHI3L1**: chitinase 3-like 1; **DENND1B**: DENN/MADD domain containing 1B; **DPP10**: dipeptidyl-peptidase 10; **ERBB2**: v-erb-b2 erythroblastic leukemia viral oncogene homolog 2; **GPI**: glycosylphosphatidylinositol; **GPI-AP: **glycosylphosphatidylinositol-anchored protein; **GSDMB**: gasdermin B; **GWAS**: genome-wide association study; **HLA**: human leukocyte antigen; **HLA-DQB1**: major histocompatibility complex, class II, DQ beta 1; **IL18R1**: interleukin 18 receptor 1; **IL1RL1**: interleukin 1 receptor-like 1; **ITGB3**: integrin beta 3; **MHC**: major histocompatibility complex; **ORMDL3**: ORM1-like 3; **PDE4D**: phosphodiesterase 4D; **PERLD1**: Per1-like domain containing 1; **RAD50**: RAD50 homolog; **SNP**: single nucleotide polymorphism; **STARD3**: StAR-related lipid transfer (START) domain containing 3; **TGFB1**: transforming growth factor beta 1.

## Competing interests

The authors declare that they have no competing interests.

## Authors' contributions

RA, AKA, PNP and BKS were involved in the design of the study, recruitment of participants for the study, and extraction of DNA samples. RA also processed and pooled the DNA samples, performed the statistical analysis and wrote the manuscript. FTC and DYW conceived, designed and planned the study and edited the manuscript. All authors have read and approved the final manuscript.

## Pre-publication history

The pre-publication history for this paper can be accessed here:

http://www.biomedcentral.com/1471-2350/12/170/prepub
